# Effects of *Lactobacillus paracei* JY062 Postbiotic on Intestinal Barrier, Immunity, and Gut Microbiota

**DOI:** 10.3390/nu17071272

**Published:** 2025-04-05

**Authors:** Jinfeng Guo, Ying Zhao, Wenqian Guo, Yilin Sun, Wei Zhang, Qianyu Zhao, Yu Zhang, Yujun Jiang

**Affiliations:** 1 Key Lab of Dairy Science, Ministry of Education, College of Food Science, Northeast Agricultural University, Harbin 150030, China; 18899535576@163.com (J.G.); 13904799723@163.com (Y.Z.); guowenqian159@163.com (W.G.); syl115630519@163.com (Y.S.); zhangwei9791@163.com (W.Z.); zhaoqy@neau.edu.cn (Q.Z.); 2 Food Laboratory of Zhongyuan, Luohe 462300, China

**Keywords:** postbiotics, intestinal barrier, intestinal inflammation, co-culture, gut microbiota

## Abstract

Background/Objectives: Research on postbiotics derived from probiotic fermented milk bases require further expansion, and the mechanisms through which they exert their effects have yet to be fully elucidated. This study utilized in vitro cell co-culture, digestion, and fermentation experiments, combined with targeted T500 technology, to elucidate the mechanism by which postbiotic Pa JY062 safeguards intestinal health. Compared to the LPS group, Pa JY062 boosted phagocytic ability in RAW264.7 macrophages, decreased NO levels, and alleviated LPS-induced excessive inflammation. Pa JY062 suppressed pro-inflammatory cytokines (IL-6, IL-17α, and TNF-α) while elevating anti-inflammatory IL-10. It prevented LPS-induced TEER reduction in Caco-2 monolayers, decreased FITC-dextran permeability, restored intestinal microvilli integrity, and upregulated tight junction genes (*ZO-1*, *occludin*, *claudin-1*, and *E-cadherin*). The hydrolysis rate of Pa JY062 progressively rose in gastrointestinal fluids in 0–120 min. At 5 mg/mL, it enriched gut microbiota diversity and elevated proportions of *Limosilactobacillus*, *Lactobacillus*, *Pediococcus*, and *Lacticaseibacillus* while augmenting the microbial production of acetic acid (120.2 ± 8.08 μg/mL), propionic acid (9.9 ± 0.35 μg/mL), and butyric acid (10.55 ± 0.13 μg/mL). Pa JY062 incorporated α_s_-casein/β-lactoglobulin hydrolysate (L-glutamic acid, alanine, lysine, tyrosine, phenylalanine, histidine, and arginine) to mitigate protein allergenic potential while harboring bioactive components, including tryptophan metabolites, vitamin B6 (VB6), and γ-aminobutyric acid (GABA). Pa JY062 represented a novel postbiotic with demonstrated intestinal health-promoting properties. These findings advance the current knowledge on postbiotic-mediated gut homeostasis regulation and expedite the translational development of dairy-derived postbiotic formulations.

## 1. Introduction

The intestine played a vital role in nutrient absorption and safeguarding systemic health. Maintaining intestinal barrier integrity is crucial for intestinal homeostasis, which comprises mechanical, chemical, microbial, and immune barriers [1]. Compromise of the mechanical barrier permitted pathogenic bacteria and their endotoxins to infiltrate the lamina propria via intestinal epithelial breaches. In severe cases, these substances entered systemic circulation, which triggered systemic inflammatory responses [2]. Functioning as the primary defense component of the intestinal barrier, the mechanical barrier comprised mucosal epithelial cells, intercellular tight junctions, adherent junctions, and desmosomes [3]. Intestinal epithelial cells modulated gut microbiota through antibacterial factor secretion and by mediating bacteria–immune cell interactions. Intestinal homeostasis relied on a balanced interplay among the microbiota, intestinal barrier, and immune system. Notably, gut immune imbalance directly contributed to the pathogenesis of inflammatory bowel diseases (Crohn’s disease and ulcerative colitis). Emerging evidence supported the therapeutic potential of microecological modulators such as probiotics [4], prebiotics [5,6], and postbiotics [7]. These interventions preserved intestinal barrier integrity and reduced inflammatory responses. Notably, microecological modulators demonstrated a favorable safety profile and efficacy without severe adverse effects.

The International Scientific Association of Probiotics and Prebiotics in 2021 defined postbiotics as the “preparation of inanimate microorganisms and/or their components that confer a health benefit on the host” [8]. Postbiotics demonstrated biological efficacy in regulating the homeostasis of the intestinal microbial ecosystem. The preservation of intestinal microbial ecosystem homeostasis relied on the collaborative actions of intestinal microorganisms, epithelial cells, stromal cells, and immune cells. Accumulating evidence has established the efficacy of postbiotics in ameliorating intestinal inflammation and preserving intestinal barrier integrity. *Lactobacillus rhamnosus* GG postbiotic HM0539 enhanced mucin 2 (MUC2) and zonula occludens-1 (ZO-1) expression and prevented dextran sodium sulfate (DSS)-induced colitis [9]. *Bifidobacterium adolescentis* B8598 postbiotics ameliorated colitis through decreased histology scores induced by DSS [10]. Batista et al. reported that heat-killed *Lactobacillus delbrueckii* CIDCA 133 and their cell-free supernatant effectively decreased the infiltration of neutrophils into the small intestine mucosa and enhanced the damaged epithelial structure caused by 5-FU in mice [11].

Our research team discovered three strains of lactic acid bacteria in previous studies demonstrating anti-inflammatory and intestinal barrier-protective effects [12,13,14]. Milk-based postbiotics were developed through process optimization combining low-temperature pasteurization with lyophilization, maximizing the retention of bioactive constituents while maintaining functional integrity. The intestinal health-promoting efficacy of postbiotics was systematically evaluated using a Caco-2/RAW264.7 co-culture model, while their digestibility, gut microbiota modulation, and short-chain fatty acid (SCFA) profiles were concurrently assessed through an integrated in vitro digestive–fermentative system. Meanwhile, the composition of postbiotics was analyzed using T500 technology, which helped to accurately understand its ingredient composition, provided strong support for product optimization and mechanism of action research, and laid the groundwork for further elucidating the mechanism through which postbiotics regulate intestinal inflammation. Furthermore, the findings offer accelerated translational opportunities across functional food development, nutraceutical production, pharmaceutical formulations, and livestock management practices.

## 2. Materials and Methods

### 2.1. Reagents and Materials

LPS and FITC-dextran (4 kDa) were obtained from Sigma-Aldrich (St. Louis, MO, USA). CCK-8 Cell Counting Kit, phosphate-buffered saline (PBS, pH 7.2), penicillin (100 U/mL), and streptomycin (100 μg/mL) were obtained from Biosharp^®^ Life Science (Hefei, China). Dulbecco’s modified Eagle medium (DMEM), high glucose, and fetal bovine serum (FBS) were acquired from Gibco (Grand Island, NY, USA). RNAiso Plus (9108), PrimeScript™ RT reagent Kit with gDNA Eraser (Perfect Real Time) (RR047A), and TB Green^®^ Premix Ex Taq™ II (Tli RNaseH Plus) (RR820A) were obtained from Takara Biomedical Technology Co., Ltd. (Beijing, China). Mouse Interleukin-6 (IL-6) ELISA Kit, Mouse Interleukin-10 (IL-10) ELISA Kit, Mouse Interleukin-17a (IL-17a) ELISA Kit, and Mouse Tumor Necrosis Factor-α (TNF-α) ELISA Kit were purchased from Xinle Biotechnology (Shanghai, China). NO kit was acquired from Beyotime (Shanghai, China). HPLC-grade acetonitrile (ACN) and methanol (MeOH) were purchased from Merck (Darmstadt, Germany). MilliQ water (Millipore, Bradford, MA, USA) was used in all experiments. All T500 standards were purchased from Sigma-Aldrich (St. Louis, MO, USA) and Shanghai Zhenzhun Biotechnology Co., Ltd (Shanghai, China). Formic acid was bought from Sigma-Aldrich (St. Louis, MO, USA). The remaining products were of analytical grade.

### 2.2. Bacterial Strains and Production of Postbiotic Powder

**Figure 1:**
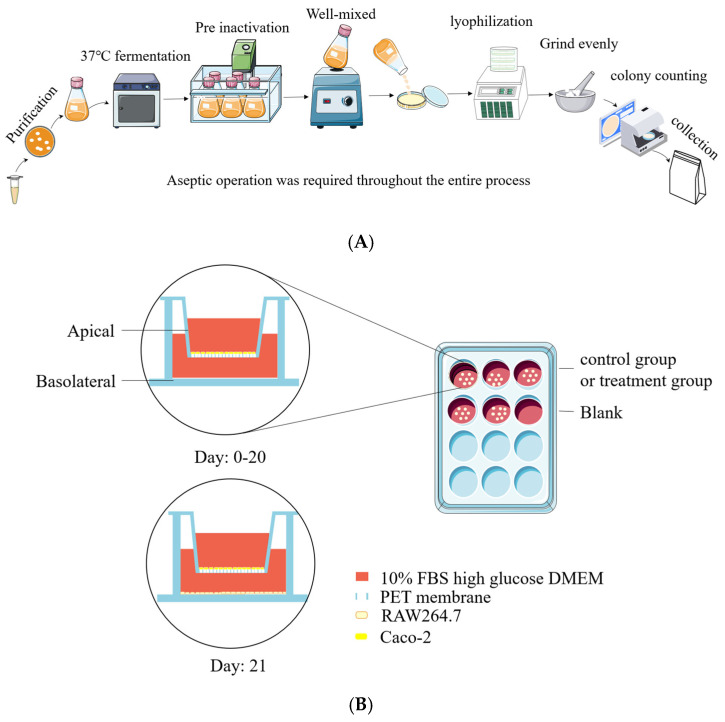
Preparation of postbiotics and cell co-culture model diagram. (**A**) Diagram illustrating preparation of postbiotics; (**B**) establishment of Caco-2/RAW264.7 co-culture system.

*Lactobacillus rhamnosus* JL-1, *Lactobacillus reuteri* J1, and *Lactobacillus paracasei* JY062 were derived from traditional fermented dairy products in Tibet, China. All strains were passed twice in de Man, Rogosa, and Sharpe (MRS) broth, centrifuged (10,000× *g*, 5 min, 4 °C), washed twice with sterile PBS (pH 7.2), and then reconstituted in skimmed milk containing 2% glucose. A bacterial suspension at 5% inoculum was cultured in skimmed milk containing 2% glucose medium, which was incubated at 37 °C with 150 rpm for 16 h. Fermentation broth with skimmed milk was heat inactivated at different temperatures and times (Table 1) and poured into a sterile glass plate, which was sealed with sterile plastic wrap and frozen at −80 °C for 24 h. Holes were pierced above each sealed plate with a toothpick, followed by lyophilization for 36 h. The lyophilized product was ground into a sterile powder, and aseptic bags were utilized and kept at 4 °C; the postbiotic suspensions were subjected to plate colony counting assay on an MRS agar plate incubated at 37 °C for 48 h. Counting results indicated that the survival rate of probiotic-derived postbiotic is 0 (Table 1). The preparation process can be seen in Figure 1A. Postbiotic preparations were designated as follows: *Lactobacillus rhamnosus* JL-1 (Re JL-1); *Lactobacillus reuteri* J1 (Rh J1); and *Lactobacillus paracasei* JY062 (Pa JY062).

### 2.3. Cell Culture and Cell Viability Assay

RAW264.7 and Caco-2 cells were derived from the Key Laboratory of Dairy Science, Ministry of Education, at the Northeast Agricultural University in China. Caco-2 cells from passages 2 to 10 were utilized, while RAW264.7 cells from passages 1 to 5 were employed due to their favorable differentiation characteristics. Caco-2 and RAW264.7 were maintained in complete cell culture medium, which contained DMEM with high glucose, 10% FBS, 1% penicillin and streptomycin, and they were incubated at 37 °C in a humidified atmosphere containing 5% CO_2_. An amount of 2 × 10^4^ cells per well were inoculated into 96-well plates for 24 h. Following this, postbiotics at varying concentrations of 50, 100, 150, 200, 250, 300, 350, 400, 450, and 500 μg/mL were introduced and cultured for an additional 24 h.

Cell Counting Kit-8 (CCK-8) was used to measure cell viability. First, the cell culture medium with postbiotics was removed and washed three times with PBS. An amount of 100 μL of fresh complete cell culture medium containing 10% CCK-8 premixed in advance was added to each well, and a well without cells was selected as a blank control. The cells were cultured for another 2 h, and the OD_450 nm_ was measured. The cell viability was calculated using the following formula:

Cell viability = [OD(drug) − OD(blank)]/[OD(0 drug) − OD(blank)] × 100%

### 2.4. Co-Culture System of Caco-2/RAW264.7 Cell

Caco-2 cells were seeded in Transwell^®^ 12-well plates (pore size 0.4 μm, Corning, NY, USA) at a density of 2 × 10^4^ cells per well and maintained in culture for a duration of 21 days to obtain a cell monolayer. Cell morphology was recorded using EVOS FL inverted fluorescence microscope at days 0, 7, 14, and 21. The sealing performance of the Caco-2 monolayer was assessed through transepidermal electrical resistance (TEER). TEER was determined as follows: the pre-alcohol-sterilized Millicell ERS volt-ohmmeter and rod probe (Bedford, MA, USA) were washed twice with PBS in a sterile cell clean bench and then carefully placed in the co-culture system. The short electrode was placed in the AP, the long electrode was placed in the BA, and the value read by the Millicell ERS volt-ohmmeter was recorded. TEER was calculated using the following formula:

TEER = (sample resistance − blank resistance) × 1.12 cm^2^

On experimental day 20, RAW264.7 macrophages were seeded in 12-well plates (1 × 10^5^ cells/well) and cultured for 24 h to establish monolayers. Subsequently, Caco-2 Transwell inserts were introduced into the RAW264.7-populated wells to establish a co-culture system [15]. Caco-2 cells cultured in Transwell^®^ inserts (12-well) were designated as the apical compartment (AP), while RAW264.7 macrophages in the basolateral chamber (BA) formed the co-culture system. The experimental setup is schematically illustrated in Figure 1B.

### 2.5. In Vitro Paracellular Permeability Assay 

The Caco-2/RAW264.7 co-culture system was employed to evaluate paracellular permeability. Postbiotics Pa JY062 (250 μg/mL) and Re JL-1 (250 μg/mL) or Rh J1 (400 μg/mL) were administered to the apical compartment (AP), whereas lipopolysaccharide (LPS, 10 μg/mL) was applied to the basolateral chamber (BA) for 12 h, with quintuplicate replicates per group. Following treatment, BA compartment media were harvested, subjected to centrifugation (2000× *g*, 5 min), and processed for inflammatory cytokine quantification. Following cell lysis of RAW264.7 in the BA for total RNA extraction, the PBS-rinsed Caco-2 monolayer was AP-loaded with 0.5 mL FITC-dextran (4 kDa, 1 mg/mL in PBS) and BA equilibrated with 1.5 mL PBS. Subsequent procedures were conducted according to the protocol by Wang et al [16].

### 2.6. Determining the Ultrastructure of the Intestinal Barrier Using Transmission Electron Microscopy

Following aspiration of culture medium from Caco-2 monolayers in 12-well Transwell^®^ plates, gentle PBS washes were performed. Triplicate wells per sample were fixed with 2.5% glutaraldehyde (4 °C, 2 h post-fixation) and trimmed into 0.2 cm × 0.2 cm specimens. Sample pieces underwent sequential processing: three 15 min washes (0.1 M PBS), 1% OsO4 stabilization (30 min), additional PBS rinses, and graded ethanol dehydration. Subsequent protocols followed established methodologies described by He et al [17]. Microvilli images were collected by transmission electron microscopy (H-7650, Hitachi, Japan).

### 2.7. Neutral Red Phagocytosis and NO Assay

RAW 264.7 macrophages were incubated in 96-well plates for 24 h. After replacing the medium with fresh complete medium containing LPS (10 μg/mL), cells were cultured for an additional 12 h. Postbiotics Pa JY062 (300 μg/mL), Re JL-1 (250 μg/mL), and Rh J1 (300 μg/mL) were then added to the medium, and cells were incubated for 24 h. One subset of cells was used for the neutral red phagocytosis assay, and another subset was used for NO detection. For phagocytosis analysis, 100 μL of 0.1% neutral red solution was added to each well, and the mixtures were incubated for 3 h. After removing the supernatant and washing with PBS, 150 μL of cell lysis buffer was added to each well. The plate was gently agitated for 15 min, and absorbance was measured at 540 nm [18].

NO assay was carried out according to the instructions provided in the NO kit.

$$\mathrm{Inhibition}\;\mathrm{rate}\;\%=\frac{1-(sampleA-blankA)}{modelA-blankA)}\times 100\%$$

### 2.8. Cellular Inflammatory Factor Assay

Mouse Interleukin-6 (IL-6) ELISA Kit, Mouse Interleukin-10 (IL-10) ELISA Kit, Mouse Interleukin-17a (IL-17a) ELISA Kit, and Mouse Tumor Necrosis Factor-α (TNF-α) ELISA Kit were employed to measure the alterations in IL-6, IL-10, IL-17a, and TNF-α levels in the cell culture supernatant of RAW264.7 cells in BA. The specific steps for collecting cell supernatants are described in detail in Section 2.5.

### 2.9. RNA Extraction and Real-Time Quantitative Polymerase Chain Reaction

Total RNA was isolated from LPS/postbiotic-treated Caco-2 monolayers and RAW264.7 macrophages using RNAiso Plus. RNA purity (1.8 ≤ A_260_/A_280_ ≤ 2.1) [19] and concentration were quantified with a NanoDrop™ ND-2000 spectrophotometer (Thermo Fisher Scientific, Waltham, MA, USA), shown in Table S1. The RNA was synthesized into cDNA using PrimeScript™ RT reagent Kit with gDNA Eraser, and the detailed process is shown in Tables S2 and S3. Real-time quantitative polymerase chain reaction (RT-qPCR) was carried out according to TB Green^®^ Premix Ex Taq™ II, and the detailed process is shown in Tables S4 and S5. RT-qPCR was used to determine relative expression levels of mRNA according to 2^−△△Ct^ method. The GAPDH was employed as a housekeeper gene. The primers used in the RT-qPCR analysis are shown in Table 2.

### 2.10. Static Simulation of Gastrointestinal Digestive Processes In Vitro

According to the INFOGEST protocol [20], PaJY062 powder was combined with ultrapure water at a 1 g:1 g ratio. A digestive chylous analog was prepared on a sterile plate using a glass rod and simulated gastric fluid (SGF) that included pepsin and gastric lipase (pH 3.0) diluted with chylous analog at a 2:2 ratio (*v*/*v*), and it was continuously stirred for 2 h to obtain gastric chyme at 37 °C at 80 rpm. Simulated intestinal fluid (SIF) including bile salts and trypsin was used after being diluted with gastric chyme at a ratio of 4:4 (*v*/*v*), adjusted to pH 7.0 with 4 M NaOH, and incubated for 2 h under 80 rpm/h for 2 h. In the stomach and intestinal simulations, a 0.5 mg/mL methanol solution of Pepstatin A was added at 0, 1, 2, 5, 10, 30, 60, and 120 min. The enzymatic reaction was halted at the conclusion of gastrointestinal digestion. Digestive juice was frozen and stored at −20 °C for subsequent analyses.

### 2.11. Degree of Protein Hydrolysis

Simulated intestinal fluid containing PaJY062 (2 mL) was mixed with trichloroacetic acid (0.75%) at a 1:1 ratio for different durations (0, 1, 2, 5, 10, 30, 60, and 120 min) and vortexed. The procedure for the simulated intestinal fluid containing PaJY06 (2 mL) was identical to that for the simulated intestinal fluid containing PaJY062(2 mL). Samples were subjected to centrifugation at 4 °C (6000 rpm/min, 20 min). The supernatant was carefully collected and filtered using a 0.45 μm membrane. An amount of 150 μL of filtrate was combined with 3 mL of o-phthalaldehyde (OPA) reagent, vortexed, and left to stand for 2 min. An amount of 200 μL of sample was transferred into a 96-well plate to assess OD_340 nm_, and ultrapure water was set as the control. Standard solutions of L-serine were prepared at concentrations of 0, 0.10, 0.20, and 0.30 mmol/L, which were reacted with OPA to create a standard curve at OD_340 nm_.

Degree of protein hydrolysis (%) = (((c × v × N)/m-β)/α)/h_tot_ × 100%

where c: the concentration obtained from incorporating the sample’s absorbance value into the serine standard curve, mmol/L; v: dilution multiples; N: volume of hydrolyzed protein solution, L; m: mass of protein involved in hydrolysis, g; α = 1; β = 0.4; h_tot_ = 8.2.

### 2.12. Simulated Gut Fermentation In Vitro

Fecal samples were collected from 10-week-old male BALB/c mice with 3% DSS-induced colitis under an ethics-approved protocol (NEAUEC20230422) by the Institutional Animal Care and Use Committee of Northeast Agricultural University. An amount of 1 mL PBS (0.1 M) was added to 1.5 mL sterile centrifuge tube containing the feces, and then the tube was vortexed and t centrifuged for 5 min (4 °C, 3000× *g*). The supernatant was aspirated into a fresh sterile microcentrifuge tube and subjected to secondary centrifugation (14,000× *g*, 5 min, 4 °C) to harvest gut microbes from the pellet. The gut microbes were resuspended in sterile modified brain heart infusion (BHI) broth for anaerobic in vitro fermentation. The modified BHI formulation was prepared according to the protocol established by Guo et al. [21]. Gut microbes were anaerobically cultured in modified BHI broth supplemented with Pa JY062 at 0, 1, 2, 3, 4, and 5 mg/mL using a 5% (*v*/*v*) inoculum. Fermentation was performed in an anaerobic chamber (N_2_:H_2_:CO_2_ = 85%:10%:5%) under 48 h of incubation at 37 °C.

### 2.13. Analysis of 16S rRNA in Gut Microbes

Gut microbe samples treated with 1, 2, 3, 4, and 5 mg/mL of PaJY062 were separately placed into centrifuge tubes and then introduced into centrifuge tubes containing the extraction lysate, and DNA was procured using the DNA Kit (D5635-02) (Omega Bio-Tek, Norcross, GA, USA). The V3-V4 segment of the gut microbes 16S rRNA gene was amplified through PCR. PCR products were quantified with the Quant-iT PicoGreen dsDNA Assay Kit and subsequently combined based on the necessary quantity for each sample. Illumina TruSeq Nano DNA LT Library Prep Kit (Personal Biotechnology, Shanghai, China) was employed to obtain library preparation. In the QIIME2 software, the number of sequences extracted at each depth and their corresponding ASV numbers were plotted to draw a rarefaction curve, depicting the microbial diversity within the community sample. Beta diversity analysis was conducted utilizing the UniFrac distance metric with R software and QIIME2 software to explore variations in microbial community structure across samples.

### 2.14. Short Chain Fatty Acids in Gut Microbes

SCFA quantification was performed via gas chromatography–mass spectrometry (GC-MS) with external standard calibration [22]. Supernatants (800 μL) from Pa JY062-treated gut microbiota cultures (1–5 mg/mL) were aliquoted into 2 mL sterile tubes, diluted to a ratio of 1:1 (*v*/*v*) with PBS, and mixed via a vortex. Diluted samples (50 μL) and 15% phosphoric acid (50 μL) were mixed, followed by the addition of internal standard solution (10 μL, 75 μg/mL isocaproic acid) and ether (140 μL). The mixture was homogenized for 1 min and then centrifuged at 4 °C (12,000 rpm/min, 10 min). The supernatant was transferred into a bottle for GC-MS analysis.

GC-MS analytical procedures were conducted following previously validated methodologies outlined by Zhang [23] and Hsu [24]. Briefly, the GC analysis was performed on trace 1310 gas chromatograph (Thermo Fisher Scientific, Waltham, MA, USA). The GC was fitted with a capillary column, Agilent HP-INNOWAX (30 m × 0.25 mm ID × 0.25 μm), and helium was used as the carrier gas at 1 mL/min. Injection was carried out in split mode at a ratio of 10:1 with an injection volume of 1 μL and an injector temperature of 250 °C. The temperatures of the ion source and MS transfer line were 300 °C and 250 °C, respectively. The column temperature was programmed to increase from an initial temperature of 90 °C, followed by an increase to 120 °C at 10 °C/min, to 150 °C at 5 °C/min, and finally, to 250 °C at 25 °C/min, which was maintained for 2 min. Mass spectrometric detection of metabolites was performed on ISQ 7000 (Thermo Fisher Scientific, Waltham, MA, USA) with electron impact ionization mode. Single ion monitoring (SIM) mode was used with the electron energy of 70 eV.

### 2.15. The Analysis of Pa JY062 Ingredients

Standard stock solutions (1 mg/mL) were prepared in methanol (MeOH) or appropriate solvents, aliquoted, and cryopreserved at −20 °C. Working solutions were generated through serial dilution with MeOH prior to analysis. Thawed Pa JY062 samples underwent brief vortex homogenization (10 sec), followed by centrifugation (12,000× *g*, 1 min, 4 °C) to ensure homogeneity. Samples (50 μL) were mixed with 250 μL of 20% (*v*/*v*) acetonitrile/methanol solution, followed by 3 min vortex homogenization and centrifugation (12,000× *g*, 10 min, 4 °C). The resultant supernatant (250 μL) was transferred to fresh tubes, incubated at −20 °C for 30 min to precipitate residual proteins, and subjected to secondary centrifugation (12,000× *g*, 10 min, 4 °C). Finally, 180 μL of clarified supernatant was filtered through a protein precipitation plate prior to LC-MS analysis. Sample extracts were analyzed using an LC-ESI-MS/MS system (UPLC, ExionLC AD, https://sciex.com.cn/; MS, QTRAP^®^ 6500+ System, https://sciex.com/). The LC-ESI-MS/MS system was accessed on 22 March 2024. The analytical conditions were as follows.

T3 method: HPLC: column, Waters ACQUITY UPLC HSS T3 C18 (100 mm × 2.1 mm i.d.′1.8 µm); solvent system, water with 0.05% formic acid (A) and acetonitrile with 0.05% formic acid (B). The gradient was started at 5% B (0 min), increased to 95% B (8–9.5 min), and finally ramped back to 5% B (9.6–12 min); flow rate, 0.35 mL/min; temperature, 40°C; injection volume, 2 μL.

Amide method: HPLC: column, ACQUITY UPLC BEH Amide (i.d. 2.1 × 100 mm, 1.7 μm); solvent system, water with 10 mM Ammonium acetate, 0.3% ammonium hydroxide (A), and 90% acetonitrile/water (*v*/*v*) (B). The gradient was started at 95% B (0–1.2 min), decreased to 70% B (8 min) and 50% B (9–11 min), and finally ramped back to 95% B (11.1–15 min); flow rate, 0.4 mL/min; temperature, 40 °C; injection volume, 2 μL. SI-MS/MS conditions were as follows: linear ion trap (LIT) and triple quadrupole (QQQ) scans were acquired on a triple quadrupole-linear ion trap mass spectrometer (QTRAP), a QTRAP^®^ 6500+ LC-MS/MS System, equipped with an ESI Turbo Ion-Spray interface, operating in both positive and negative ion modes and controlled using Analyst 1.6.3 software (Sciex). The ESI source operation parameters were as follows: ion source, ESI+/−; source temperature, 550 °C; ion spray voltage (IS), 5500 V (positive) and −4500 V (negative); and curtain gas (CUR), 35 psi. Metabolites were analyzed using scheduled multiple reaction monitoring (MRM). Data acquisitions were performed using Analyst 1.6.3 software (Sciex). Multiquant 3.0.3 software (Sciex) was used to quantify all metabolites. Mass spectrometer parameters, including the declustering potentials (DPs) and collision energies (CEs) for individual MRM transitions, were determined with further DP and CE optimization. A specific set of MRM transitions were monitored for each period according to the metabolites eluted within this period.

### 2.16. Statistical Analysis

All experimental procedures were conducted with ≥3 biological replicates, with exact repetition numbers specified in corresponding figure legends. Statistical analyses were performed using IBM SPSS Statistics 25.0 (Armonk, NY, USA), with data expressed as mean ± SD. TEER temporal profiles were analyzed using a repeated-measures ANOVA (RM-ANOVA), while other parameters were evaluated via a one-way ANOVA with Bonferroni-adjusted post hoc comparisons.

## 3. Results

### 3.1. Postbiotics Promoted Cell Viability in a Manner Dependent on Dosage

**Figure 2:**
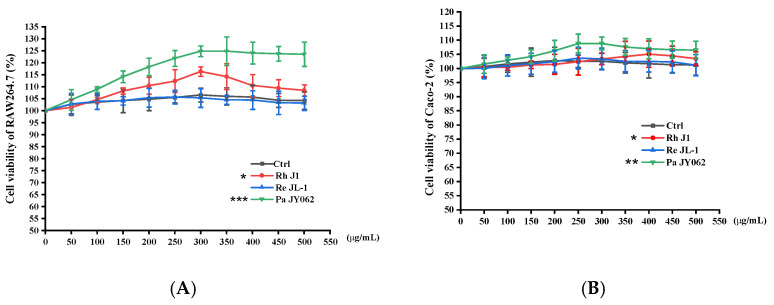
Dose-dependent effects of postbiotics on cellular viability after 24 h treatment. (**A**) Viability of RAW264.7 macrophages; (**B**) viability of Caco-2 intestinal epithelial cells. Postbiotics were tested at concentrations of 50–500 μg/mL. Data represent mean ± SD (*n* = 5 biological replicates). Statistical significance versus untreated controls: * p < 0.05, ** p < 0.01, *** p< 0.001.

A cell viability assessment revealed that postbiotics dose-dependently enhanced RAW264.7 cell viability versus the untreated controls (Ctrl), with Pa JY062 (300 μg/mL) exhibiting the most pronounced enhancement (*p* < 0.001, Figure 2A). Notably, postbiotic treatments maintained Caco-2’s proliferative capacity (Figure 2B), showing maximal viability at 250 μg/mL of Pa JY062. Based on these findings, Pa JY062 and Rh J1 were prioritized for subsequent mechanistic investigations.

### 3.2. The Effect of Postbiotics on Intestinal Barrier Integrity

**Figure 3:**
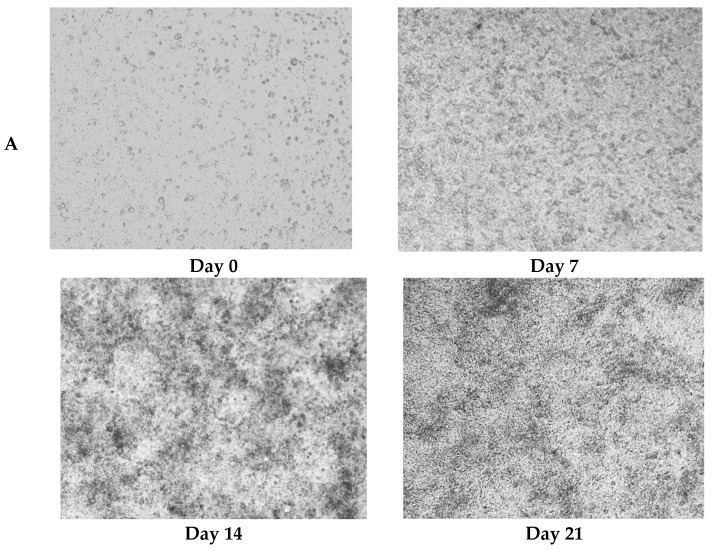

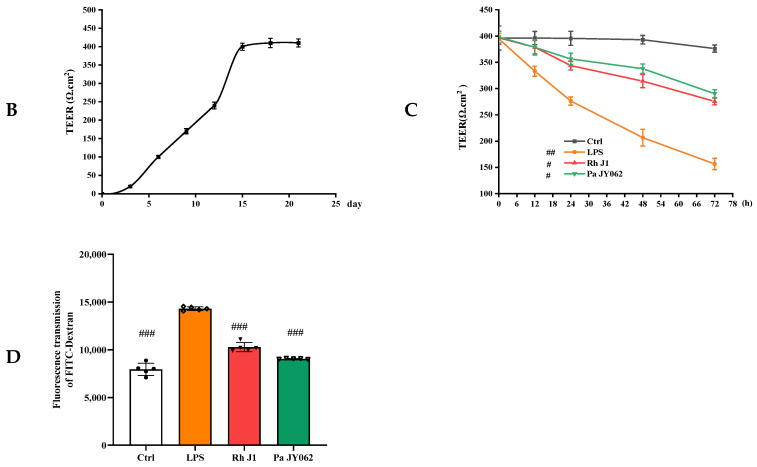
Protective effects of postbiotics on intestinal barrier integrity. (**A**) Morphological evolution of Caco-2 monolayers during differentiation (20× magnification); (**B**) trans epithelial electrical resistance (TEER) dynamics during 21-day monolayer maturation (days 0, 7, 14, and 21); (**C**) TEER modulation by postbiotics in LPS-induced barrier dysfunction; (**D**) FITC-dextran paracellular flux quantification in LPS-challenged monolayers. Data are presented as mean ± SD (*n* = 5); # *p* < 0.05, ##*p*<0.01, ### *p* < 0.001 vs. LPS group. Different shapes in the figure represent different samples, and the same shape represents a repetition of the sample.

Caco-2 monolayers recapitulated morphological and functional hallmarks of intestinal epithelial differentiation [25]. During the 21-day differentiation period (days 0–21), Caco-2 cells progressively transitioned from dispersed single cells to confluent polarized monolayers, ultimately establishing a mature epithelial barrier characterized by tight junction integrity and brush border formation (Figure 3A).

TEER, quantified by ionic flux through the paracellular pathway, served as a quantitative biomarker of monolayer integrity and tight junction maturation [26]. The attainment of TEER ≥ 400 Ω·cm^2^ (day 15: 400.0 ± 9.6 Ω·cm^2^) confirmed the establishment of functional barrier competence [27], with subsequent stabilization (days 18–21) demonstrating robust intestinal barrier maturation through Caco-2 differentiation (Figure 3B).

To assess the barrier protective efficacy of postbiotics against LPS-induced intestinal barrier dysfunction, TEER of Caco-2 monolayers was monitored at 0, 12, 24, 48, and 72 h post-treatment. LPS challenge caused severe barrier disruption, reducing TEER by 238.2 ± 0.35 Ω·cm^2^ over 72 h (*p* < 0.001; Figure 3C). Notably, Pa JY062 exhibited the most pronounced barrier protective efficacy, limiting TEER reduction to 107.93 ± 3.64 Ω·cm^2^ (*p* < 0.001 vs. LPS group), indicative of preserved paracellular permeability and tight junction integrity.

To further analyze the effects of postbiotics on intestinal barrier integrity, paracellular permeability was assessed by measuring FITC-dextran across Caco-2 monolayers. As shown in Figure 3D, both Rh J1 and Pa JY062 significantly attenuated LPS-induced barrier disruption, with Pa JY062 demonstrating the most potent protective efficacy (*p* < 0.01).

### 3.3. The Effect of Postbiotics on the Intestinal Barrier Microstructure

**Figure 4:**
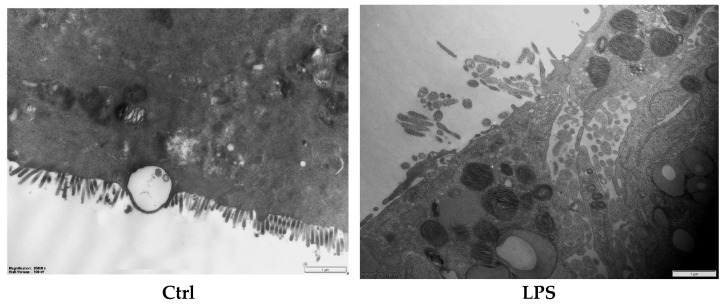

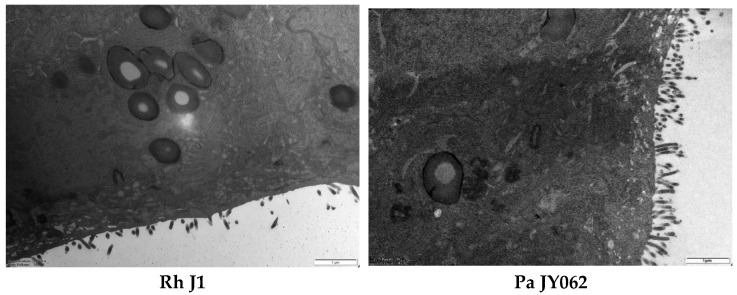
The effect of postbiotics on the ultrastructure in LPS-induced intestinal epithelial barrier dysfunction (magnification: ×20,000; *n* = 3).

Microvilli are critical ultrastructural components of the intestinal epithelium; they can maintain epithelial barrier integrity and regulate paracellular permeability [28]. Transmission electron microscopy revealed distinct morphological differences: the control (Ctrl) monolayers exhibited densely packed microvilli with organized brush borders, whereas treated LPS displayed severe loss of microvilli and widened intercellular spaces accompanied with a disordered manner (Figure 4). Postbiotic intervention restored the density and structural organization of microvilli across all groups, with Pa JY062 demonstrating the most significant microvillus morphology.

### 3.4. The Influence of Postbiotics on the mRNA Expression of Intestinal Barrier Protein

**Figure 5:**
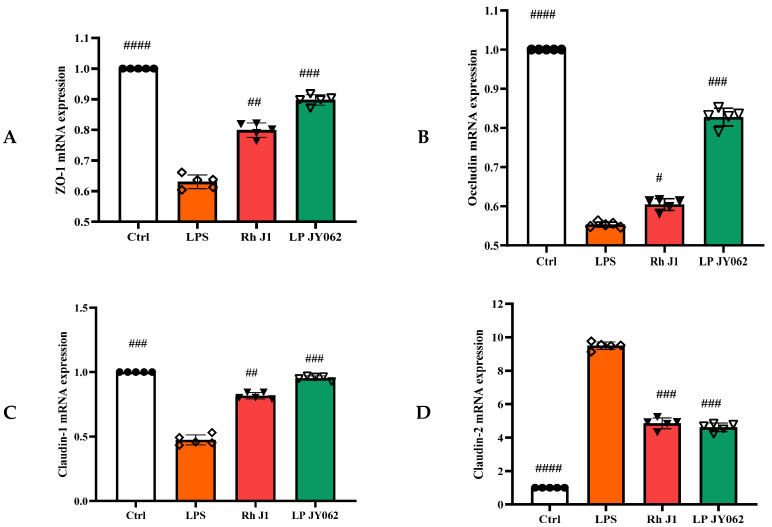

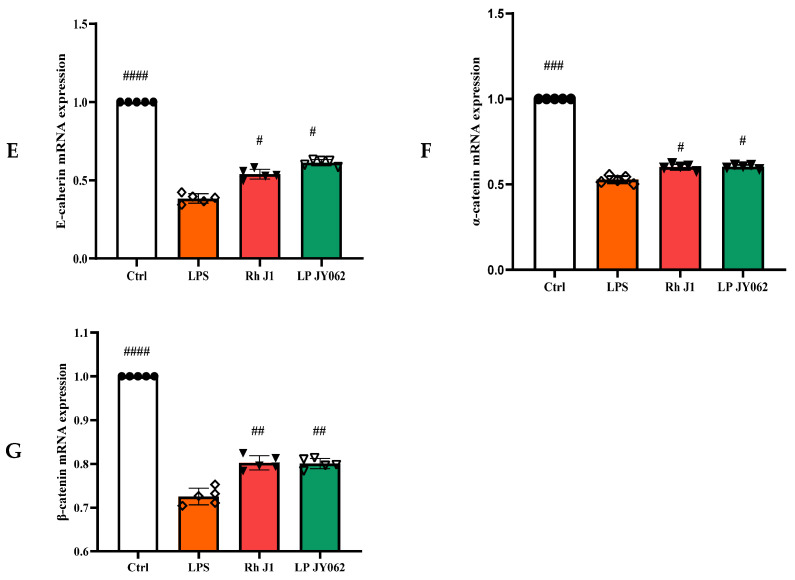
The effect of postbiotics on the relative mRNA expression of tight junctions (*ZO-1*, *occludin*, *claudin-1*, and *claudin-2*) and adhesive junctions (*E-cadherin*, *α-catenin*, and *β-catenin*) in LPS-induced intestinal epithelial barrier dysfunction. (**A**) *ZO-1*; (**B**) *occludin*; (**C**) *claudin-1*; (**D**) *claudin-2*; (**E**) *E-cadherin*; (**F**) *α-catenin*; and (**G**) *β-catenin*. The values are expressed as the mean ± SD (*n* = 5); # *p* < 0.05, ## *p* < 0.01, ### *p* < 0.001, and #### *p* < 0.0001 vs. the LPS. Different shapes in the figure represent different samples, and the same shape represents a repetition of the sample.

To comprehensively analyze the impact of postbiotic intestinal barrier injury, we conducted the mRNA expression of tight junction proteins (*ZO-1*, *occludin*, *claudin-1*, and *claudin-2*) and adhesive junction proteins (*E-cadherin*, *α-catenin*, and *β-catenin*). Postbiotics significantly increased the mRNA expression of *ZO-1*, *occludin*, and *claudin-1* (Figure 5A−C) compared to the LPS group; the mRNA expression of *claudin-2* (Figure 5D) was significantly decreased; and the Pa JY062 group showed the most significant regulation effect (*p* < 0.001). Meanwhile, postbiotics successfully reversed the decrease in *E-cadherin* (Figure 5E) mRNA expression caused by LPS, with Pa JY062 showing the best effect compared to the LPS group. Postbiotic intervention showed a trend toward upregulating the mRNA expression of *α-catenin* (Figure 5F) and *β-catenin* (Figure 5G) compared to the LPS group, though these alterations remained statistically non-significant (*p* > 0.05).

### 3.5. The Influence of Postbiotics on Intestinal Inflammation

**Figure 6:**
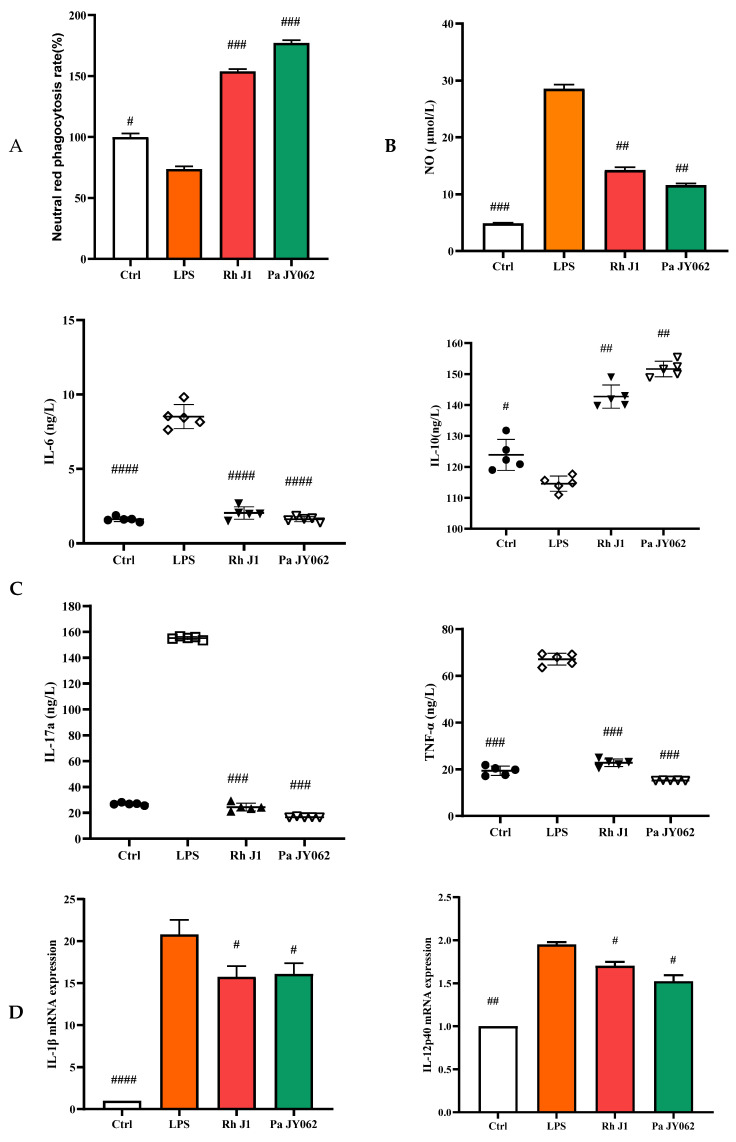

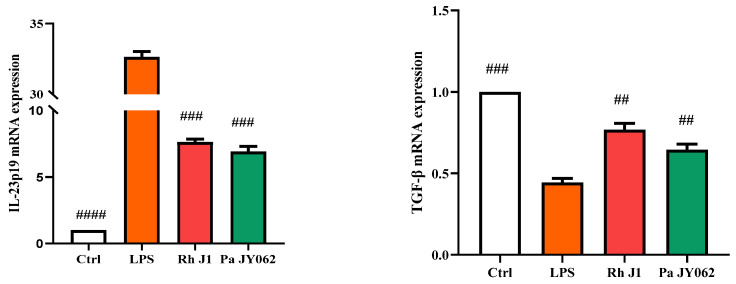
The influence of postbiotics on intestinal inflammation. (**A**) The impact of postbiotics on RAW264.7 cells based on the rate of neutral red phagocytosis. (**B**) The impact on the NO content of postbiotics. (**C**) The impact of postbiotics on RAW264.7 cells based on the contents of IL-6, IL-10, IL-17a, and TNF-α. (**D**) The impact of postbiotics on the mRNA expression of IL-1β, IL-12p40, IL-23p19, and TGF-β. The values are expressed as the mean ± SD (*n* = 5); # *p* < 0.05, ## *p* < 0.01, ### *p* < 0.001, and #### *p* < 0.0001 vs. the LPS. Different shapes in the figure represent different samples, and the same shape represents a repetition of the sample.

The compromise of the intestinal barrier resulted in immune dysfunction within the gut, consequently exacerbating the onset of intestinal inflammation. To provide a more comprehensive understanding of the influence of postbiotics on intestinal health, we employed a co-culture model of Caco-2 and RAW264.7 cells to investigate the effects of postbiotics on the interplay between intestinal epithelial cells and immune cells. Pa JY062 significantly improved the phagocytic ability (*p* < 0.001, Figure 6A) and reduced the production of NO (*p* < 0.01, Figure 6B) in RAW264.7 compared to the LPS group. Pa JY062 reduced the contents of IL-6, IL-17a, and TNF-α and enhanced the content of IL-10 (Figure 6C). Pa JY062 reduced the mRNA expression of *IL-1β*, *IL-12p40*, and *IL-23p19* while increased the mRNA expression levels of *TGF-β* (Figure 6D).

### 3.6. Effects of PaJY062 on Protein Digestion in In Vitro Simulated Digestion Model

**Figure 7:**
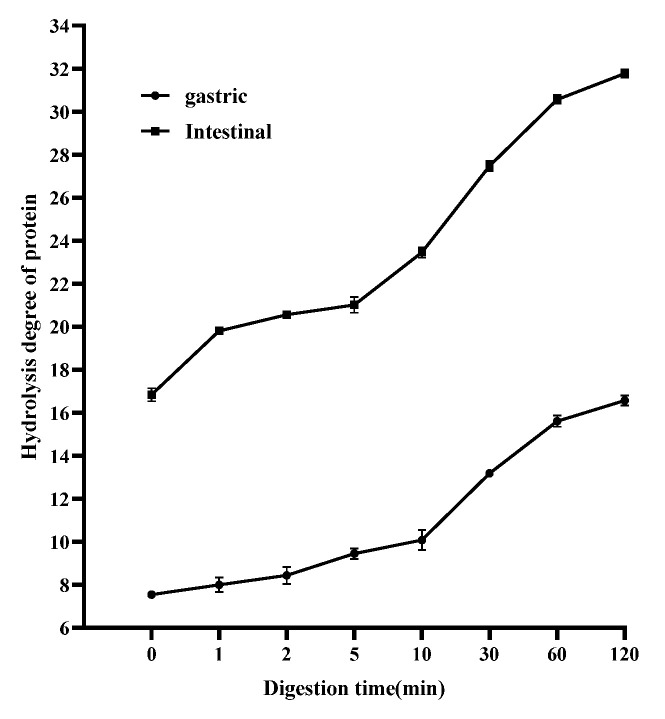
Changes in proteolysis rate of postbiotics over 0–120 min of in vitro digestion. Values are expressed as mean ± SD (*n* = 3).

The degree of hydrolysis of PaJY062 gradually increased in gastric juice over a period of 0 to 120 min (an increase of 9.02%), whereas it rose by 14.94% in intestinal juice, suggesting that PaJY062 can be effectively utilized, particularly in the intestine (Figure 7). Good protein hydrolysis resulted in the production of more easily digestible small peptides and amino acids (see the ingredients of Pa JY062 for details).

### 3.7. Effect of PaJY062 on Gut Microbiota and SCFAs

**Figure 8:**
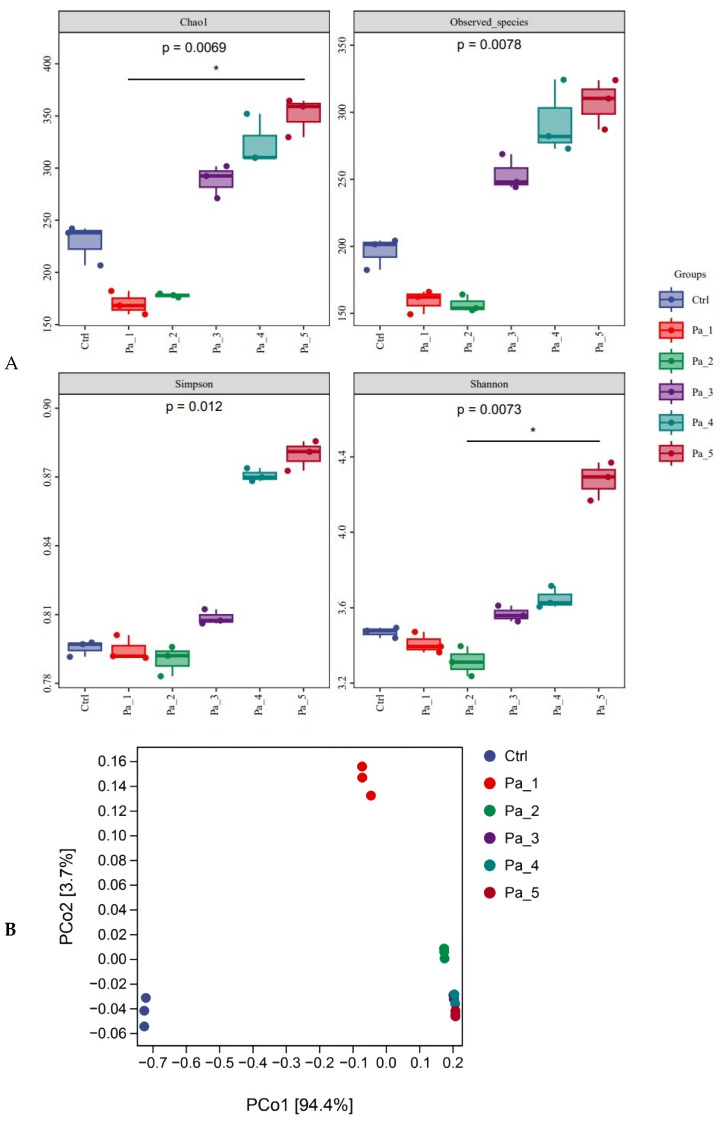

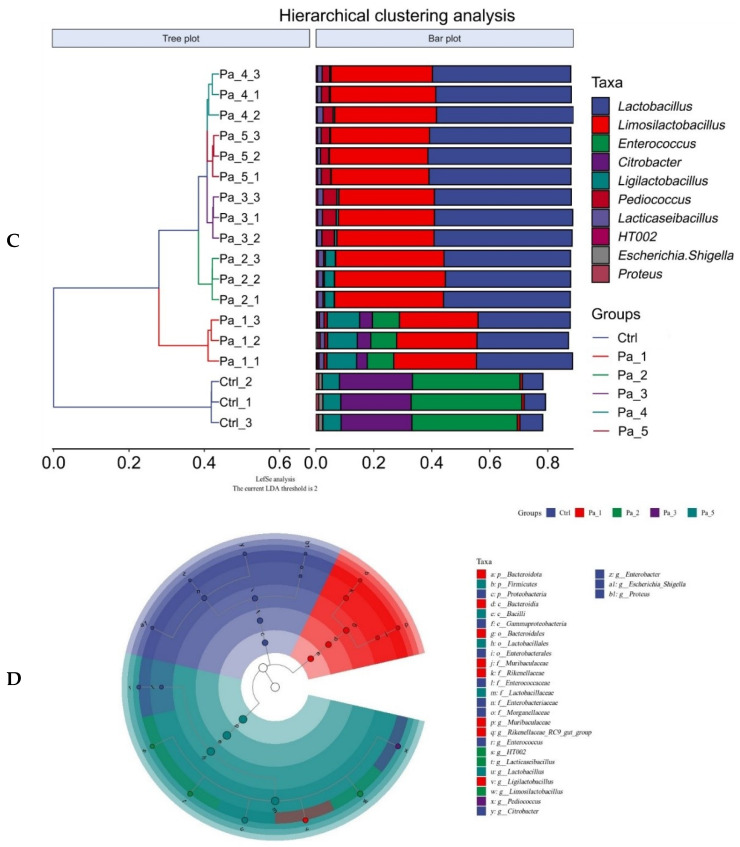

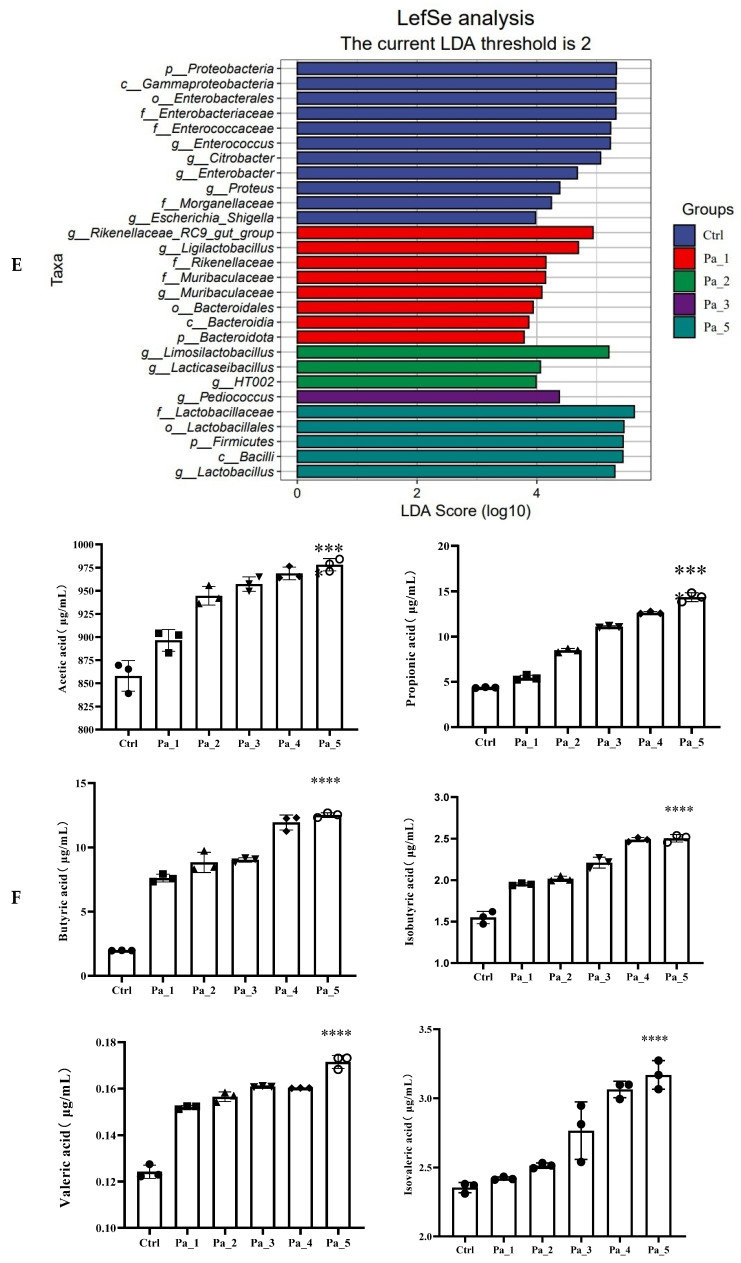

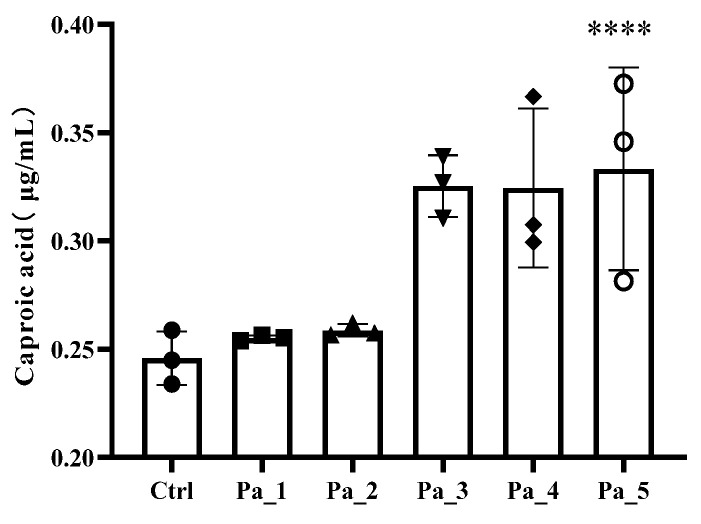
Effect of change in different concentrations of PaJY062 on gut microbiota and production of SCFAs. (**A**) Alpha diversity index; (**B**) beta diversity; (**C**) composition species; (**D**) linear discriminant analysis (LDA); (**E**) LDA Effect Size (LEfSe) analysis; (**F**) change in SCFAs. Values are presented as mean ± SD (*n* = 3). * *p* < 0.05, *** *p* < 0.001 and **** *p* < 0.0001 vs. Ctrl. Different shapes in the figure represent different samples, and the same shape represents a repetition of the sample.

After gastrointestinal digestion, PaJY062 was further fermented in vitro using mice feces to investigate its influences on the intestinal microbiota of colitis mice. Alpha diversity typically encompassed Chao1 and the observed species indices, which delineated richness, along with the Shannon and Simpson indices, which delineated diversity [29]. Chao1 and the observed species indices increased at 5 mg/mL (Pa_5) of PaJY062, suggesting that PaJY062 could improve the species richness of the intestinal microbiota of mice suffering from colitis. For the Shannon and Simpson indices at Pa_5, PaJY062 exerted a more pronounced influence on the α diversity of the gut microbiota in mice afflicted with colitis (Figure 8A). Beta diversity refers to the differences in species composition among various communities across environmental gradients; thus, it is also called between-habitat diversity [29]. The PCoA diagram illustrates that the distances between Ctrl, Pa_1, and Pa_2 are quite substantial, indicating significant beta diversity among them, while the overlap among Pa_3, Pa_, and Pa_5 indicate that they are similar in beta diversity (Figure 8B). As PaJY062 was introduced, there was a noticeable shift in the composition of intestinal species in colitis mice. It was observed that compared to the Ctrl, the proportion of *Limosilactobacillus*, *Lactobacillus*, *Pediococcus*, and *Lacticaseibacillus* in the in vitro fermentation of intestinal microbiotas increased progressively with the rising concentration of PaJY062. The proportion of *Enterococcus*, the opportunistic pathogen *Citrobacter*, and the harmful bacteria *Escherichia shigella* decreased (Figure 8C).

To identify the specific groups that were significantly influenced by PaJY062, linear discriminant analysis (LDA) and LDA Effect Size (LEfSe) analysis were performed, with the results presented in Figure 8D,E. The LDA score bar positively correlates with the relative abundance of discriminative bacterial taxa. It was observed that the abundance of *Ligilactobacilus*, *Limosilactobacillus*, *Lactobacillaceae*, *Lactobacillaceae*, and *Lactobacillus* increased with the addition of PaJY062.

The SCFA concentrations exhibited dose-dependent elevations with an increasing Pa JY062 dosage, reaching maximal levels in the Pa_5 group (Figure 8F). Notably, PaJY062 had the greatest effect on the acetate concentration, increasing it by 120.19 ± 6.53 μg/mL compared with 857.97 ± 19.67 μg/mL in the control group, which was consistent with the diversity of the intestinal microbiota. Simultaneously, the incorporation of PaJY062 exerted a considerable influence on the propionic acid and butyric acid levels.

### 3.8. PaJY062 Contained a Variety of Beneficial Ingredients

The components of Pa JY062 are elaborated on in Table 3, wherein 14 substances exhibited a concentration exceeding 100,000 ng/mL in addition to the metabolites of tryptophan (specifically, 3-indolactic acid, indole-3-carboxaldehyde, tryptophol, pyridoxal hydrochloride, arginine, and γ-aminobutyric acid). Furthermore, our findings reveal the presence of hydrolysis products of α_s_-casein and β-lactoglobulin, such as L-glutamic acid, alanine, lysine, tyrosine, phenylalanine, histidine, and arginine, within PaJY062.

## 4. Discussion

Inflammatory bowel disease (IBD) and related intestinal pathologies are pathologically defined by compromised intestinal epithelial barrier integrity and macrophage hyperactivation. The Caco-2 intestinal epithelial model has been extensively employed for investigating intestinal pathophysiology, drug transport mechanisms, and colitis-related pathogenesis. Concurrently, RAW264.7 macrophages serve as pivotal mediators of innate immunity through the secretion of immunoregulatory cytokines that orchestrate adaptive immune activation. Our optimized co-culture system recapitulates disease-relevant cellular interactions while overcoming the physiological limitations of monoculture systems, providing a cost-efficient platform for simulating in vivo microenvironments.

The current study was predicated on the Caco-2/RAW264.7 intestinal model induced by LPS to systematically investigate the anti-inflammatory properties of postbiotics and their protective effects on the intestinal barrier. TEER reflected the integrity of tight junctions and the overall barrier function among epithelial cells. A higher TEER value signified a more robust barrier function and reduced permeability. FITC-dextran (4 kDa) is closer to physiological molecules that are small in size and can more easily pass through damaged barriers. TEER and FITC-dextran permeability can accurately reflect the changes in barrier function under different physiological or pathological conditions. We observed an imbalance in the interaction between Caco-2 cells and RAW264.7 macrophages, leading to sustained inflammation and tissue damage, providing insights into the disease and potential therapeutic targets. Our findings indicate that PaJY062 exhibited no toxicity towards RAW264.7 and Caco-2 cells, and it effectively alleviated the LPS-induced increase in TEER and the FITC-dextran permeability of the intestinal barrier. Similarly to the effects of PaJY062, *Lactobacillus plantarum* 1.0386 postbiotics could improve transepithelial electrical resistance enhance and mitigate the disruption of paracellular permeability, and enhance the tight junction in Caco-2 [30]. Heat-killed *Enterococcus faecium* BGPAS1-3 possessed the capability to protect against tight junction disruption in differentiated Caco-2 monolayers challenged by *L. monocytogenes* ATCC 19111 [31]. Postbiotic muramyl dipeptide can decrease apoptosis and mitigate increases in intestinal barrier paracellular permeability by LPS exposure in Caco-2 cells [28]. Postbiotics, compared with the LPS/pathogen, demonstrated effective preservation of intestinal barrier integrity in Caco-2 models by elevating TEER and mitigating FITC-dextran permeability.

The protective effect of postbiotics on the intestinal barrier was further evidenced by their capacity to restore microvillus architecture. We also observed that the microvilli of the intestinal barrier progressively regained their brush-like structure and tight junctions under the influence of PaJY062. The restoration of the brush border structure promoted the recovery of the absorption function of intestinal epithelial cells, enhanced nutrient absorption capacity, helped rebuild the intestinal barrier function, and reduced the invasion of pathogens. A similar phenomenon was noted in postbiotics 1.0386-Slp [32]. Tight junctions regulated cell bypass permeability and maintained cell polarity. The adhesive junction was located below the tight junction of epithelial cells, which enabled the connection between two cells by interacting with E-cadherin, α, β, and γ-catenin [33,34]. Postbiotics (butyrate and active vitamin D3) enhanced the expression of *ZO-1* in the cecum of mice infected with *Salmonella* [35]. However, the expression of claudin did not exhibit a significant decrease in Caco-2 cells infected with *Listeria monocytogenes* ATCC 19111. The mRNA expression of occludin was regulated by heat-killed *Enterococcus faecium* BGPAS1-325. The fermentation of cow’s milk using *Lactobacillus paracasei* CBAL74 demonstrated a protective effect that enhanced occludin and ZO-1 expression and significantly enhanced E-cadherin expression on Caco-2 cells [36]. Consistent with other postbiotics, Pa JY062 demonstrated efficacy in enhancing the stability of the intestinal barrier and in managing intestinal inflammatory diseases caused by LPS. These findings from different postbiotics suggest that postbiotics have the potential to maintain intestinal health by protecting the intestinal barrier.

Impaired integrity of the intestinal barrier triggered a cascade of intestinal inflammation. This study revealed that Pa JY062 significantly reduced the release of pro-inflammatory cytokines, including IL-6, IL-17a, TNF-α, IL-1β, IL-12p40, and IL-23p19, while simultaneously enhancing the production of IL-10 and TGF-β, as well as augmenting the phagocytic capacity of RAW264.7 cells. These findings are consistent with previous studies that *Saccharomyces boulardii* and postbiotics significantly diminished the release of pro-inflammatory factors such as IL-1β, IL-6, IL-17a, and TNF-α induced by DSS while enhancing the secretion of IL-10 [37]. The collaboration between TNF-α, IL-1, and IL-6 facilitated the migration of immune cells to the site of inflammation. The overexpression of IL-17a elicited the production of TNF-α, IL-6, and IL-1β, thereby exacerbating the severity of inflammation. Conversely, a reduction in IL-17a diminished the synthesis of these inflammatory mediators while simultaneously modulating Treg/Th17 balance by augmenting the proportion of Treg cells. Treg cells alleviated inflammation by inhibiting the activity of effector T cells and indirectly by secreting anti-inflammatory factors IL-10 and TGF-β, hence inhibiting the inflammatory response. IL-23 shared a common p40 subunit with IL-12 [38], while the p19 subunit specifically influenced IL-23. IL-12 stimulated the Th1 response, whereas IL-23 promoted the Th17 response; both disrupted intestinal homeostasis, accelerated inflammation, and played an essential role in the progression of intestinal inflammation. Heat-killed *Companilactobacillus crustorum* MN047 substantially reversed TNF-α and IL-6 in DSS induced colitis mice [39]. *Limosilactobacillus reuteri* DS0384 protected intestinal epithelium subjected to injury induced by IFNγ and TNFα in a human intestinal organoid model [40]. In summary, therapeutic targeting of the Th17/Treg immune axis was a key strategy to improve intestinal inflammation, and postbiotics regulated this immune network through multiple targets, providing a potential solution for the treatment of inflammatory diseases associated with intestinal barrier damage.

Given that the anti-inflammatory effect of Pa JY062 was closely related to its gastrointestinal digestion and absorption and its effect on intestinal flora, we next analyzed the effects of Pa JY062 on the intestinal microbiota and short-chain fatty acids in vivo; this research also encompassed in vitro digestion and fermentation experiments. Pa_5 (PaJY062, 5 mg/mL) exerted a better influence on the α diversity of the gut microbiota, especially for the growth of beneficial bacteria, such as *Limosilactobacillus*, *Lactobacillus*, *Pediococcus*, and *Lacticaseibacillus. Limosilactobacillus*, *Lactobacillus*, *Pediococcus,* and *Lacticaseibacillus* produced lactic acid, acetic acid, lactobacillus, and other substances through in vivo fermentation, which played the role of inhibiting pathogenic bacteria. This finding is consistent with Mu’s research, which found that postbiotics have more *Lactobacillus*, *Lactobacillus* sp. ASF360, and *Lactobacillus vaginalis* in the fecal microbiota of groups M and H [41]. Through the restoration of the increased abundance of *Lactobacillus* and *Rikenellaceae*, along with a decrease in the prevalence of *Turicibacter* and *Escherichia-Shigella*, *Saccharomyces boulardii* BR14 mitigated colitis [42]. The ADR-159 postbiotics exerted a beneficial effect on inflammation induced by *Citrobacter* [43]. *Lactobacillus parabuchneri* postbiotics MF2103 enriched the diversity of microbiota within the *Faecalibacterium* enterotype [44], while Guo et al. found that postbiotic intervention not only increased beneficial gut bacteria such as *Dysosmobacter welbionis* and *Faecalibacterium prausnitzii*, resulting in elevated concentrations of acetic acid, propionic acid, butyric acid, caproic acid, and valeric acid, but also reduced potential pathogens like *Megamonas funiformis* [45]. This evidence indicates that postbiotics reshaped gut microbes, promoted the production of SCFAs, and then regulated the immune balance and barrier function, thereby achieving the initial effect of alleviating intestinal inflammation such as colitis.

Furthermore, a detailed analysis of the specific components of Pa JY062 was conducted to elucidate which constituents contribute to its protective role in intestinal health-promoting properties. PaJY062 comprises a variety of beneficial components, including metabolites of tryptophan (3-indolactic acid, indole-3-carboxaldehyde, and tryptophol), pyridoxal hydrochloride, arginine, and γ-aminobutyric acid. Studies showed that 3-indolactic acid (IAA) mitigated DSS-induced colitis in mice by modifying the gut microbiome. In the group treated with IAA, there was a notable increase in the abundance of *Bifidobacterium pseudolongum* [46]. 3-carboxaldehyde effectively protected against colon length shortening and damage induced by DSS in the colon, notably reducing the severity of inflammation. The production of inflammatory factors of TNF-α, IL-6, and IL-1β was significantly reduced under 3-IAld treatment. 3-carboxaldehyde provided effective protection against the shortening of the colon length and damage caused by DSS, significantly diminishing the severity of inflammation. The production of inflammatory factors such as TNF-α, IL-6, and IL-1β was markedly reduced when treated with 3-IAld both in vivo and in vitro [47]. Samantha A. Scott discovered that three gut microbially synthesized compounds—indole-3-ethanol, indole-3-pyruvate, and indole-3-aldehyde—enhance the integrity of the intestinal barrier and provide protection against inflammation associated with IBDs [48]. Pyridoxal hydrochloride (V_B6_) intake alleviated IBD in mice [49]. The expression of Arg1 was found to be associated with the level of inflammation in intestinal tissues from patients with IBD [50]. Furthermore, our findings reveal the presence of hydrolysis products of α_s_-casein and β-lactoglobulin, such as L-glutamic acid, alanine, lysine, tyrosine, phenylalanine, histidine, and arginine, within PaJY062. This suggests that the allergenic components found in milk are transformed into easily absorbable amino acids via fermentation. Consequently, PaJY062 offers therapeutic benefits for patients suffering from ulcerative colitis.

While acknowledging the inherent limitations of in vitro models in replicating physiological complexity (e.g., neuroendocrine regulation, host-microbiome crosstalk, and systemic immune interactions), we initiated complementary in vivo validation studies using a dextran sulfate sodium (DSS)-induced ulcerative colitis model to validate Pa JY062′s therapeutic efficacy under physiologically relevant conditions.

Despite these limitations, we demonstrated that *Lactobacillus paracasei* JY062 postbiotics could effectively sustain intestinal health. Mechanistically, our findings indicate that this protective effect primarily arose from the presence of small-molecule active ingredients with anti-inflammatory activity and good absorption, which collaboratively functioned to regulate inflammation, bolster the epithelial barrier, and modulate the intestinal microbiota and short-chain fatty acids, thereby enhancing the structure of the colonic mucosa. *Lactobacillus paracasei* JY062 postbiotic is a skimmed milk-based postbiotic that can be well digested and absorbed by the human body. This positions it as a promising therapeutic agent for the alleviation of intestinal inflammation.

## 5. Conclusions

This study illustrates that the postbiotic Pa JY062 can effectively enhance the intestinal health of the Caco-2/RAW264.7 co-culture system by alleviating LPS-induced intestinal barrier damage and intestinal inflammation. In particular, it reduces the FITC-dextran permeability of the intestinal barrier, restores the microvillus structure, and enhances the mRNA expression levels of *ZO-1*, *claudin-1*, *occludin* and *E-cadherin*. It promotes the restoration of Th17/Treg balance by reducing pro-inflammatory factors and enhancing the secretion of anti-inflammatory factors, and it also promotes the increase in beneficial intestinal bacteria and SCFAs. Its intestinal protective effect is mainly due to its presence of multiple bioactive components, such as tryptophan metabolites, GABA, VB6, etc. This study provides scientific value for the widespread application of more probiotic-derived postbiotics by clarifying the beneficial effects of PaJY062 in the intestine. This study provides a theoretical basis for the widespread application of probiotic-derived postbiotics. However, current research is still mainly focused on in vitro studies, and the physiological complexity of the host cannot be fully simulated, which may limit the widespread application of the commercialization process of postbiotics. Therefore, we need to further conduct in vivo studies on Pa JY062 to alleviate colitis to accelerate the further application and promotion of Pa JY062 in the field of intestinal health.

## Figures and Tables

**Table 1:** The conditions for the postbiotics.

	*Lactobacillus reuteri* J1 (CFU/mL)	*Lactobacillus rhamnosus* JL-1 (CFU/mL)	*Lactobacillus paracasei* JY062 (CFU/mL)
65 °C 30 min before	1.36 × 10^12^	1.32 × 10^13^	2.42 × 10^12^
65 °C 30 min after	100	3400	35,000
70 °C 15 min before	1.32 × 10^12^	1.35 × 10^13^	2.45 × 10^12^
70 °C 15 min after	60	350	1500
70 °C 30 min before	1.31 × 10^12^	1.36 × 10^13^	2.61 × 10^12^
70 °C 30 min after	0	120	320
75 °C 15 min before	1.39 × 10^12^	1.44 × 10^13^	2.51 × 10^12^
75 °C 15 min after	0	40	50
75 °C 30 min before	-	1.35 × 10^13^	2.51 × 10^12^
75 °C 30 min after	-	0	0
lyophilization before	1.36 × 10^12^	1.32 × 10^13^	2.42 × 10^12^
lyophilization after	500	3800	1500
70 °C 15 min and lyophilization before	1.31 × 10^12^	1.36 × 10^13^	2.61 × 10^12^
70 °C 15 min and lyophilization after	0	20	86
70 °C 30 min and lyophilization before	-	1.33 × 10^13^	2.52 × 10^12^
70 °C 30 min and lyophilization after	-	0	0

Note: - indicates that this step was not implemented.

**Table 2:** Specific primer sequences.

Primer Name	Forward Primer (5′-3′)	Reverse Primer (5′-3′)
*ZO-1*	TGAGGCAGCTCACATAATGC	GGTCTCTGCTGGCTTGTTTC
*Occludin*	AAAGGGCATTGCTCATCCTGA	ACAATGGCAATGGCAATTCATC
*Claudin-1*	CCAGTCAATGCCAGGTACGAAT	GGCCTTGGTGTTGGGTAAGA
*Claudin-2*	CCATGGTCAACACAACAGCA	GGCATCTAGAAGACCTGAATGG
*E-cadherin*	CCCAAACGTAACGAGGGTATC	GGCAGCTTGAAGTGGTAGAAGT
*α-catenin*	CAACCCTTGTAAACACCAAT	ACTGAACCTGACCGTACACCTTCTCCAAGAAATTCTCA
*β-catenin*	CGCTTGGCTGAACCATCACA	AGCAGCTTTATTAACTACCACCT
*IL-1β*	GGACAGGATATGGAGCAACAAGTGG	TCATCTTTCAACACGCAGGACAGG
*IL-12 p40*	AGACCCTGCCCATTGAACTG	CAGGAGTCAGGGTACTCCCA
*IL-23 p19*	AGAGCCAGCCAGATYTGAGAAG	CTGCTCCRTGGGCAAAGA
*TGF-β*	TACAGCAACAATTCCTGGCGATACC	CTCAACCACTGCCGCACAACTC
*GAPDH*	F: GAGAAGGCTGGGGCTCATTT	TAAGCAGTTGGTGGTGCAGG

**Table 3:** Components of in Pa JY062.

Substance	Substance Type	Chemical Formula	Content (ng/mL)
Dihydroxyacetone phosphate	Phosphoric acids	C_3_H_7_O_6_P	1,199,244
N-Acetyl-D-Galactosamine	Amines	C_8_H_15_NO_6_	457,323.6
α-Ketoglutaric Acid	Organic acid and its derivatives	C_5_H_6_O_5_	384,721.2
rac Normetanephrine Hydrochloride	Hormones and hormone-related compunds	C_9_H_14_ClNO_3_	275,776.8
N-Acetylneuraminic Acid	Amino acid derivatives	C_11_H_19_NO_9_	274,082.4
2-Methyllactic acid	Organic acid and its derivatives	C_4_H_8_O_3_	248,861.4
Pantothenic acid	Sugars	C_9_H_17_NO_5_	245,755.8
L-Pyroglutamic acid	Amino acids	C_5_H_7_NO_3_	157,103.4
L-Glutamic acid	Amino acids	C_5_H_9_NO_4_	150,073.8
Choline alfoscerate	LPC	C_8_H_20_NO_6_P	140,098.2
Glutaric acid	Organic acid and its derivatives	C_5_H_8_O_4_	118,312.2
L-Leucic acid	Amino acids	C_6_H_12_O_3_	117,873
L-Alanine	Amino acids	C_3_H_7_NO_2_	107,682
N-Acetyl-L-glutamic acid	Amino acid derivatives	C_7_H_11_NO_5_	104,548.2
Indolelactic acid	Indole and its derivatives	C_11_H_11_NO_3_	3846.162
Indole-3-Carboxaldehyde	Indole and its derivatives	C_9_H_7_NO	18.5718
Tryptophol	Indole and its derivatives	C_10_H_11_NO	438.7266
Pyridoxal hydrochloride	CoEnzyme and vitamins	C_8_H_10_ClNO_3_	2699.274
Arginine	Amino acids	C_6_H_14_N_4_O_2_	19,382.1
γ-Aminobutyric acid	Organic acid and its derivatives	C_4_H_9_NO_2_	59,846.52
L-Glutamic acid	Amino acids	C_5_H_9_NO_4_	150,073.80
Lysine	Amino acids	C_6_H_14_N_2_O_2_	13,724.82
Tyrosine	Amino acids	C_9_H_11_NO_3_	77,773.2
Phenylalanine	Amino acids	C_9_H_11_NO_2_	81,640.2
Histidine	Amino acids	C_6_H_9_N_3_O_2_	85,330.8

## Data Availability

The data in the project are still being collected, but all data used in this study are available by contacting the authors.

## Supplementary Materials

The following supporting information can be downloaded at: https://www.mdpi.com/article/10.3390/nu17071272/s1, Table S1: RNA purity and concentration; Table S2: Genomic DNA removal reaction; Table S3. Reverse transcription reaction; Table S4: PCR reaction system; Table S5: Real-Time PCR Reaction Procedure.

## Abbreviations

The following abbreviations are used in this manuscript:

LPS	Lipopolysaccharide
Pa JY062	*Lactobacillus paracei* JY062 postbiotics
Re JL-1	*Lactobacillus rhamnosus* JL-1 postbiotics
V_B6_	Pyridoxal hydrochloride
GABA	γ-aminobutyric acid
Rh J1	*Lactobacillus reuteri* J1 postbiotics
IL-6	Interleukin-6
IL-17a	Interleukin-17a
TNF-α	Tumor necrosis factor-α
IL-10	Interleukin-10
NO	Nitric oxide
TEER	Transmembrane electrical resistance
FITC	Fluorescein 5-isothiocyanate
mRNA	Messenger RNA
ZO-1	Zonula occludens-1
MUC2	Mucin 2
DSS	Dextran sodium sulfate
SCFAs	Short-chain fatty acids
kDa	Kilodalton
PBS	Phosphate-buffered saline
DMEM	Dulbecco’s modified Eagle medium
FBS	Fetal bovine serum
ELISA	Enzyme-linked immunosorbent assay
MRS	Man, Rogosa, and Sharpe
AP	Apical side
BA	Basal side
TEM	Transmission electron microscopy
OD	Optical density
cDNA	Complementary DNA
RT-qPCR	Real-time quantitative polymerase chain reaction
GAPDH	Glyceraldehyde-3-phosphate dehydrogenase
SGF	Simulated gastric fluid
SIF	Simulated intestinal fluid
OPA	O-phthalaldehyde
ASV	Amplicon sequence variant
GC-MS	Gas chromatograohy-mass spectrometry
LC-MS/MS	Liquid chromatography–tandem mass spectrometry
ANOVA	One-way analysis of variance
Ctrl	Control
TGF -β	Transforming growth factor-β
IL-1β	Interleukin-1β
IL-12p40	Interleukin-12 p40 subunit
IL-23p19	Interleukin-23 p19 subunit
Th1	helper T cell 1
Th2	helper T cell 2
LDA	linear discriminant analysis
LEfSe	LDA effect size
IBD	Inflammatory bowel disease
