# Supplementary material for: Effects of Lactobacillus paracei JY062 Postbiotic on Intestinal Barrier, Immunity, and Gut Microbiota
Source: Nutrients. 2025 Apr 5;17(7):1272. doi: 10.3390/nu17071272 (PMC11990213; doi:10.3390/nu17071272)
Supplement: Supplementary file 1 [file nutrients-17-01272-s001.zip › nutrients-3511076-supplementary.pdf]

**Table S1.** RNA purity and concentration.

| Samples | purity (A260/A280) | concentration（ng/μL） |
|---|---|---|
| Ctrl-1 | 2.02 | 803 |
| Ctrl-2 | 1.98 | 931 |
| Ctrl-3 | 1.96 | 1027 |
| Ctrl-4 | 1.97 | 1715 |
| Ctrl-5 | 1.91 | 1204 |
| LPS-1 | 1.95 | 1505 |
| LPS-2 | 1.93 | 1565 |
| LPS-3 | 1.98 | 1820 |
| LPS-4 | 1.91 | 1388 |
| LPS-5 | 2.08 | 1362 |
| Rh J1-1 | 1.97 | 865 |
| Rh J1-2 | 1.93 | 1661 |
| Rh J1-3 | 2.07 | 1321 |
| Rh J1-4 | 1.91 | 1833 |
| Rh J1-5 | 1.95 | 1998 |
| Pa JY062-1 | 1.89 | 1011 |
| Pa JY062-2 | 1.96 | 1541 |
| Pa JY062-3 | 1.99 | 1271 |
| Pa JY062-4 | 1.92 | 966 |
| Pa JY062-5 | 1.93 | 1931 |

As demonstrated in Supplementary Figure S1, the RNA purity of all samples ranged from 1.9 to 2.1, indicating the absence of protein and phenolic contamination, thus confirming high purity. The RNA concentration measured exceeded 500 ng/μL, signifying a very high concentration that could be appropriately diluted for further use.

cDNA synthesis conditions include two parts: (1) Genomic DNA removal reaction; (2) Reverse transcription reaction.

(1) **Genomic DNA removal reaction**. The reaction mixture was prepared on ice according to the following ingredients. To ensure the accuracy of the reaction mixture preparation, for each reaction, the Master Mix was prepared based on the number of reactions plus two, then vortexed and mixed, dispensed10 μL into each reaction tube, and finally, the RNA sample was added. A PCR instrument was used to incubate under the following conditions: 42°C for 2 min, 4°C ∞.

**Table S2.** Genomic DNA removal reaction

| Reagents | Dosage |
|---|---|
| **5×gDNA Eraser Buffer** | 2.0 μL |
| **gDNA Eraser** | 1.0 μL |
| **Total RNA** | *1 |
| **RNase Free dH$_2$O** | up to 10 μL |

*1： In a 20 μL reverse transcription reaction system, TB Green qPCR can use up to 1 μg of total RNA.

To ensure the accuracy of the reaction solution preparation, the Master Mix was prepared based on the number of reactions plus two for each reaction. After the reaction solution was prepared on ice, it was gently mixed. Then, 10 μL of the Master Mix was transferred into each genomic DNA removal reaction tube, mixed gently, and the reverse transcription reaction was carried out immediately. A PCR instrument was used to incubate under the following conditions: 37℃ 15 min, 85℃ 5 sec, 4℃ ∞.

**Table S3.** Reverse transcription reaction

| Reagents | Dosage | |
|---|---|---|
| genomic DNA removal reaction solution | 10.0 μl | Master Mix |
| PrimeScript RT Enzyme Mix I | 1.0 μl | |
| RT Primer Mix | 1.0 μl | |
| 5×PrimeScript Buffer 2（for Real Time ) | 4.0 μl | |
| RNase Free $dH_2O$ | 4.0 μl | |
| | Total 20 μl | |

**Table S4.** PCR reaction system (the reaction solution was prepared on ice, applied Biosystems 7300/7500 Real-Time PCR System)

| Reagents | Dosage | Final concentration |
|---|---|---|
| **TB Green Premix Ex Taq Il(Tli RNaseH Plus)(2×)** | 10 μl | 1× |
| **PCR Forward Primer(10 μM)** | 0.8 μl | 0.4 μM*1 |
| **PCR Reverse Primer(10 μM)** | 0.8 μl | 0.4 μM*1 |
| **ROX Reference Dye or Dye I II (50×) *3** | 0.4 μl | |
| **RT reaction solution (cDNA solution)** | 2 μl | |
| **RNase Free $dH_2O$** | 6 μl | |
| **Total** | 20 μl | |

**Table S5.** Real-Time PCR Reaction Procedure

| steps | programme | Cycle times | Temperature | Time |
|---|---|---|---|---|
| **Stage 1** | Pre-denaturation | 1 | 95 ℃ | 30 s |
| **Stage 2** | PCR reaction | 40 | 60 ℃ | 34 s |
| **Melting curve Stage** | | 1 | 95 ℃ | 15 s |
| | | 1 | 60 ℃ | 1 min |
| | | 1 | 95 ℃ | 15 s |
